# Factors Influencing Fertility Distress in Reproductive-Aged Gynecologic Cancer Patients: A Narrative Overview

**DOI:** 10.3390/jcm14238512

**Published:** 2025-11-30

**Authors:** Jing Deng, Jing Chen, Xiujing Guo, Chuan Xie

**Affiliations:** 1 Department of Gynecology and Obstetrics Nursing, West China Second University Hospital, Sichuan University, Chengdu 610041, China; 2 Key Laboratory of Birth Defects and Related Diseases of Women and Children (Sichuan University), Ministry of Education, Chengdu 610041, China; 3 Department of Gynecology and Obstetrics, West China Second University Hospital, Sichuan University, Chengdu 610041, China

**Keywords:** reproductive-age, gynecological cancers, fertility distress, influencing factors, psychosocial

## Abstract

**Objective**: To synthesize the multifactorial determinants of fertility distress among reproductive-aged women with gynecological cancers, elucidating complex interactions among biological, psychological, and sociocultural dimensions. **Methods**: Comprehensive literature searches were performed in PubMed, Embase, and PsycINFO to identify relevant studies on factors influencing fertility distress in gynecologic cancer patients. **Results**: Gynecological cancers pose significant threats to women’s health, particularly in terms of fertility protection, which is a key priority for patients of childbearing age. Individuals diagnosed with these malignancies frequently experience fertility-related distress stemming from physiological alterations and exacerbated by psychological factors. Moreover, sociocultural support systems, familial expectations, and cultural norms significantly influence reproductive decision-making and psychological adjustment in this population. Fertility distress is determined by complex interactions among these dimensions. **Conclusions**: By integrating biological, psychological, and sociocultural perspectives, we provide clinical guidance for developing targeted interventions that address patients’ comprehensive needs, ultimately advancing patient-centered oncological care.

## 1. Introduction

Gynecological cancers are increasingly recognized as a significant global health concern, with current epidemiological trends showing a consistent rise in both incidence and mortality rates, particularly in low- and middle-income countries (LMICs) [1]. According to the World Health Organization’s International Agency for Research on Cancer (IARC), the global cancer burden is projected to increase by 77% by 2050, with a rising trend in gynecologic malignancies, further amplifying the importance of addressing fertility-related issues in this population [2]. Notably, a concerning trend has emerged, the incidence of endometrial carcinomas was 20–25% and the incidence of epithelial ovarian cancers was 3–17% occurring among women of reproductive age (under 40 years) [3]. However, with the proportion of primipregnancy women aged 30 and older rising from 11% to 26% between 2000 and 2020, many patients are diagnosed with cancer before they fulfill their reproductive goals [4].

**Figure 1:**
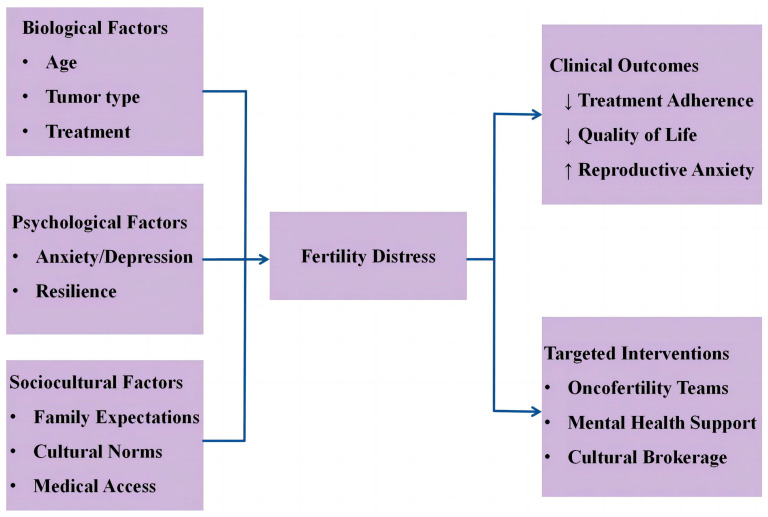
Integrative Framework of Fertility Distress. Arrow directions denote the change in measures: upward (↑) for increase and downward (↓) for decrease.

Conventional therapies for gynecological cancers, including surgical resection, radiation therapy, and chemotherapy, can all affect fertility [5]. Fertility distress refers to young women’s concerns about cancer treatment-induced or potential future reproductive impairment and child-rearing challenges [6]. Evidence suggests that these concerns may outweigh disease-related anxieties for many patients [7]. These women often experience psychological distress associated with uncertainty about fertility preservation options, anticipatory grief regarding potential reproductive loss, and changes in body image. These psychological factors can significantly influence treatment adherence and overall quality of life [8]. Fertility distress encompasses physiological factors, familial and sociocultural dimensions. The aim of this study is to elucidate the complex interactions among biological, psychological, and sociocultural factors, providing guidance for clinicians in developing targeted interventions to meet the comprehensive needs of these patients (Figure 1).

## 2. Methods

For this narrative review, a literature search was performed in PubMed, Embase, and PsycINFO from inception to January 2025. The search strategy incorporated terms for gynecologic cancers (e.g., “ovarian cancer”, “cervical cancer”, “endometrial cancer”) and fertility-related distress (e.g., “reproductive distress”, “psychological distress”). The final search was executed on 31 January 2025. Retrieved records were screened for studies examining factors linked to fertility concerns in reproductive-aged gynecologic cancer patients. Non-original articles such as commentaries, letters, and conference abstracts were excluded.

This narrative overview synthesizes factors based on their established clinical relevance and recurrent empirical evidence in the literature concerning fertility distress in gynecologic cancer. The selected factors represent core determinants across the biopsychosocial spectrum:

(1) Biological factors, including age, tumor type, and cancer treatment methods, were included for their direct, mechanistic impact on ovarian function and fertility potential, forming the non-negotiable biological foundation of distress.
(2) Psychological factors, particularly the patient’s psychological state (e.g., anxiety, depression), were prioritized due to their well-documented role as both outcomes and amplifiers of fertility-related distress.
(3) Socio-cultural and structural factors encompassing the impact of family and socioeconomic status, accessibility to medical resources, and cultural background shape the perception of fertility and the social pressures encountered by a patient.

This review did not extend to broader macro-level factors (e.g., national health policies) or highly specific biological markers (e.g., genetic profiles), as these fall outside its focused scope on the immediate biopsychosocial experience of the individual patient.

**Figure 2:**
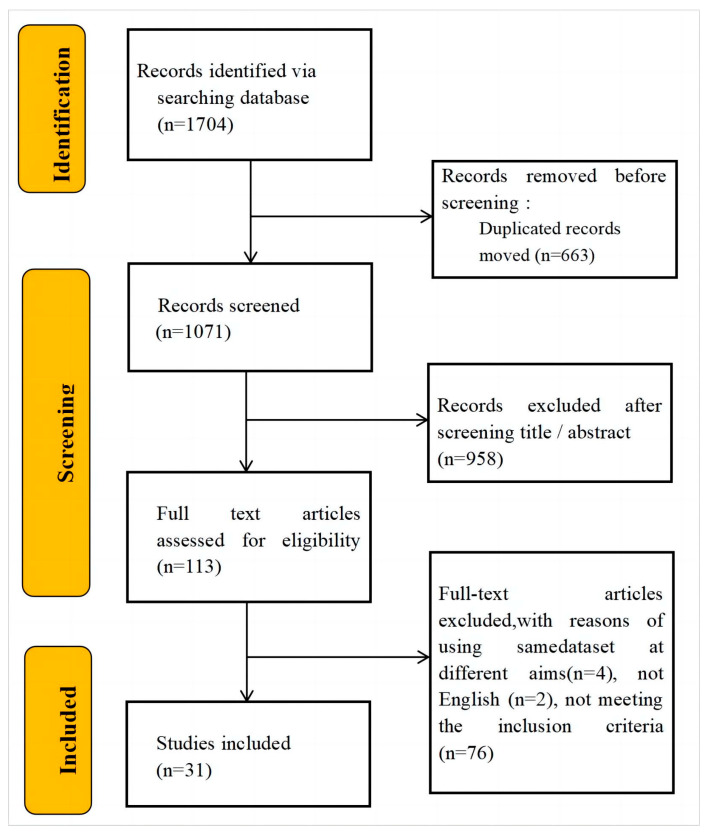
Flow chart outlining the literature search and selection process.

A total of 1704 articles were identified by searching databases. Following duplicate removal, we screened 1071 records by title and abstract, subsequently selecting 113 articles for full-text assessment. Ultimately, 31 studies met predefined eligibility criteria. A flow diagram outlines the study selection process to ensure transparency (Figure 2).

## 3. Biological Factors

### 3.1. The Impact of Age on Fertility

Age is a significant biological factor that influences female fertility, resulting in a gradual decline in both the quantity and quality of oocytes, which subsequently leads to a significant decrease in live birth rates [8]. Rising cancer incidence in women of reproductive age, particularly those without children, is increasing clinical focus on fertility preservation for this group [9]. Age is regarded as a critical factor influencing fertility outcomes among cervical cancer patients [10]. Moreover, advancing age is associated with an increased risk of reproductive health conditions such as polycystic ovary syndrome and endometriosis, both of which are prevalent among women of reproductive age and may further complicate fertility [11]. This dual role of age as both a biological clock and a risk factor for other gynecological conditions emphasizes its pivotal role in fertility preservation. Considering these biological realities, it is imperative to conduct comprehensive, age-specific assessments when formulating fertility preservation strategies, ensuring that personalized evaluations of risks and benefits account for the multifaceted ways in which aging impacts reproductive health outcomes (Table 1).

### 3.2. The Relationship Between Tumor Type and Fertility Distress

The relationship between tumor type and fertility distress is a complex and clinically significant aspect of cancer treatment. It exhibits considerable variability across different malignancies. Primary gynecological cancers, such as ovarian and endometrial cancers, can directly compromise the structure and function of reproductive organs through local invasion and damage to surrounding tissues. In contrast, cancers originating outside the reproductive system (e.g., breast and thyroid cancers) primarily impact fertility indirectly. This occurs primarily through systemic treatment effects like chemotherapy, hormonal therapies, and radiation [12]. Patients with breast or cervical cancer frequently experience heightened fertility distress, stemming from both direct ovarian damage resulting from treatment and associated hormonal imbalances. Notably, women with uterine fibroids, despite the benign nature of these growths, often encounter substantial challenges in conceiving following treatment [13]. Patients with ovarian cancer may experience direct damage to ovarian function during treatment, potentially leading to infertility [14]. On a more positive note, benign tumors treated with fertility-preserving methods generally have minimal adverse effects on reproductive health [15]. Understanding these nuances is critical for developing personalized treatment strategies that allow healthcare providers to create effective fertility preservation plans tailored to the specific characteristics of each tumor (Table 1).

### 3.3. The Impact of Cancer Treatment Methods on Fertility Distress

Gynecological cancers are typically managed with surgery, often supplemented by chemotherapy and radiotherapy. Surgical removal of the uterus or ovaries directly impacts fertility, frequently resulting in infertility [16]. Chemotherapy and radiotherapy both damage reproductive cells, reducing oocyte production and quality and this effect is especially significant in younger patients [17]. Chemotherapeutic agents such as cyclophosphamide and doxorubicin are well-documented for significantly suppressing gonadal function, leading to reduced fertility and an increased risk of premature menopause in women following treatment [18]. A study on breast cancer patients revealed that those undergoing combined chemotherapy and endocrine therapy report heightened fertility anxiety [19]. Meanwhile, radiotherapy can directly damage reproductive organs, resulting in ovarian dysfunction. Patients receiving radioiodine therapy often express concerns about potential ovarian impairment and its implications for subsequent lactation [20].

Consequently, it is necessary to assess both the efficacy of treatment and its associated fertility risks, engaging in detailed discussions with patients regarding fertility evaluation and preservation strategies. This approach supports informed consent for cancer treatment decisions. It also allows customization of fertility preservation plans, balancing oncological care with reproductive autonomy (Table 1).

## 4. Psychosocial Factors

### 4.1. The Impact of Psychological State on Fertility Distress

Psychological morbidity has a profound impact on health trajectories and quality of life. Anxiety and depression are often more prevalent, particularly in populations managing chronic conditions or fertility challenges. For women undergoing repeated unsuccessful fertility preservation attempts, reproductive struggles can trigger emotional distress, intensifying psychological burden and affecting both physiological health and treatment efficacy in turn [21]. For example, disruptions to fertility care led to significant psychological distress and anxiety for many patients during the COVID-19 pandemic [22]. Research further demonstrates an inverse relationship between psychological resilience and fertility-related concerns in reproductive-aged cancer patients [23]. Those with lower resilience experience heightened anxiety about treatment-induced fertility impairment.

Patients undergoing fertility preservation usually experience significantly higher levels of anxiety compared with the general population, often accompanied by depressive symptoms [24]. These psychosocial factors interact in complex ways, collectively influencing the effectiveness of fertility preservation efforts [25]. Psychological disorders have been shown to directly correlate with treatment outcomes [26]. Accordingly, routine psychological assessment and evidence-based mental health interventions should be integrated into standard fertility preservation protocols. This is essential to optimize psychosocial support and improve quality of life throughout care (Table 2).

### 4.2. The Role of Social Support Systems

#### 4.2.1. The Impact of Family and Socioeconomic Status on Fertility Distress

Social support involves the assistance from family members and resources provided by professional institutions. It is a critical component of care for patients facing health-related challenges. A strong support system is conducive to markedly reducing symptoms of anxiety and depression, enhancing life satisfaction [27]. It also improves the coping capacity of both patients and family members who provide emotional and practical assistance during the fertility preservation process [28]. For instance, family support significantly alleviates psychological distress and bolsters confidence in achieving reproductive goals among young patients with cervical cancer who pursue fertility preservation [29]. Spousal relationship is especially influential in this context. Emotional closeness and mutual understanding can mitigate fertility-related anxiety and depression, enabling couples to navigate reproductive challenges together [30]. Better marital intimacy is associated with fewer fertility distress, whereas partner conflict may intensify anxiety and adversely affect mental health [31].

Additionally, socioeconomic status is a critical determinant of fertility-related decision-making and health outcomes. Individuals and families generally have better access to medical care and greater autonomy in making reproductive choices because of their higher socioeconomic status. For example, young adults from economically advantaged backgrounds are more likely to leverage a wide range of social resources and prioritize family planning and prenatal care [32]. In contrast, those with limited financial means face elevated fertility risks and disproportionately adverse health outcomes [32]. In many low- and middle-income countries, financial barriers frequently impede access to essential reproductive health services [33]. Efforts to reduce socioeconomic disparities and strengthen social support networks are essential to enhancing psychological resilience and improving health outcomes among women pursuing fertility preservation (Table 2).

#### 4.2.2. The Impact of Accessibility to Medical Resources on Fertility Distress

Access to medical resources is a fundamental social determinant that significantly influences individual health outcomes and reproductive choices. In resource-limited or geographically isolated areas, constrained healthcare access can severely hinder individuals’ ability to obtain essential care, thereby influencing fertility decisions and overall health outcomes [34]. The difficulty in acquiring medical information is crucial in determining the quality of fertility decisions and patients’ satisfaction. The use of digital platforms to disseminate health information can enhance patient engagement in treatment decisions and improve overall satisfaction [35]. Moreover, timely consultations regarding fertility preservation following treatment can facilitate informed family planning and alleviate psychological stress [36]. Multidisciplinary approaches that combine fertility counseling with educational support not only meet the informational needs of young gynecological cancer patients considering fertility preservation but also improve health literacy and reduce fertility distress [37].

Despite recent advancements in medical technology, significant disparities continue to exist. Many patients encounter barriers to accessing post-treatment fertility information, driven by the uneven distribution of medical resources, gaps in clinician expertise, and cognitive challenges at the patient level [38]. As a result, inadequate fertility counseling often leaves individuals feeling underinformed and unsupported when making complex reproductive decisions, particularly in regions where information accessibility is limited. To ensure that patients receive comprehensive guidance and can exercise autonomy in their fertility decisions, healthcare institutions need to expand fertility consultation services and the enhancement of provider expertise in oncofertility. These initiatives are essential for improving the success rates of fertility preservation and alleviating the psychosocial burdens associated with fertility (Table 2).

## 5. The Impact of Cultural Background on Fertility

The cultural background of patients with gynecologic cancer profoundly influences the nature and severity of their fertility-related distress. Cultural norms not only shape reproductive values but also define the social and familial pressures that can intensify psychological distress after a cancer diagnosis. In Asian and African societies, where fertility is often regarded as a fundamental duty linked to a woman’s identity and family legacy, patients may experience distress marked by deep-seated guilt, shame, and the burden of meeting collective familial obligations [39,40]. In some Muslim communities, fertility is viewed as a “divine blessing” deeply intertwined with family and societal responsibilities [41,42]. By contrast, in Western individualistic cultures, greater emphasis is placed on personal autonomy, life planning, and reproductive self-determination. Here, distress often revolves more around the loss of personally envisioned futures, disruption of identity, and frustration over limited choices due to illness [43]. This cultural lens has critical clinical implications. Recognizing whether a patient’s distress arises primarily from external family expectations or from internal grief over lost autonomy is key to delivering culturally competent psychosocial care. As globalization progresses, assisted reproductive technologies (ART) add another dimension of cross-cultural variation [44,45], where disparities in access and acceptance may either alleviate or amplify a patient’s distress [46]. These evolving landscapes underscore the adaptability of cultural values, providing valuable insights for updating fertility policies and rethinking social structures in response to changing reproductive realities (Table 3).

## 6. Future Research Directions—Exploration and Implementation of Intervention Studies

Fertility distress stems from a complex mixture of biological, psychological, social, and cultural factors. To address these issues effectively, future research and clinical practices should focus on integrative assessment frameworks that can guide personalized intervention strategies. One promising area is the use of digital health technologies, which offer innovative ways to conduct intervention research. For instance, remote monitoring can facilitate customized interventions that boost patient engagement and adherence, ultimately improving the effectiveness of these interventions [47]. Furthermore, it is essential to conduct long-term follow-ups on intervention outcomes to continuously assess patients’ quality of life, providing comprehensive guidance for clinical practice [48]. These approaches reduce fertility worries while improving physical and mental health. This improvement enhances patients’ overall wellbeing (Table 4).

## 7. Study Limitations

This overview synthesizes the multifactorial nature of fertility distress in gynecologic cancer, though several limitations should be noted. First, limited examination of partner dynamics exists despite evidence that spousal relationships significantly influence distress levels, with partner conflict intensifying anxiety and adversely affecting mental health. Second, inadequate exploration of cultural contexts is evident, particularly regarding how traditional views (e.g., fertility as a “divine blessing” or “fundamental duty”) interact with modern reproductive technologies across diverse societies. Finally, the relationship between fertility distress and more severe, chronic psychological sequelae, particularly post-traumatic stress disorder (PTSD), requires deeper investigation. Although evidence directly linking PTSD to fertility distress remains limited, the trauma of a cancer diagnosis and treatment may interact bidirectionally with fertility concerns. Future longitudinal and mixed-methods studies are essential to clarify this complex interplay.

## 8. Clinical Implications

This review has the following clinical implications: (1) Implement multidisciplinary fertility preservation teams involving oncologists, fertility specialists, and psychologists to concurrently address biological factors (age-specific fertility preservation strategies and tumor type-specific interventions), psychological distress (treatment-related anxiety, anticipatory grief, and body image concerns), and sociocultural dimensions (familial expectations and cultural norms). (2) Incorporate routine psychological assessment and evidence-based mental health interventions into standard fertility preservation protocols to optimize psychosocial support and improve quality of life, given the significant impact of psychological morbidity on treatment efficacy. (3) Develop accessible resources and expand fertility consultation services using digital platforms to disseminate health information, enhance patient engagement, and address socioeconomic barriers and regional disparities in healthcare access.

## 9. Conclusions

Fertility distress in reproductive-aged gynecologic cancer patients manifests through complex, multifactorial pathways. Biological, psychological, social, and cultural determinants interact dynamically across the disease trajectory, from diagnosis through treatment to survivorship, significantly shaping reproductive decisions, mental health, and quality of life. This review delineates these intricate interrelationships and underscores their clinical implications. Future research should prioritize developing evidence-based, multidisciplinary reproductive planning strategies. Such interventions should target modifiable factors to mitigate fertility-related distress and enhance holistic wellbeing in this vulnerable population.

## Figures and Tables

**Table 1:** Biological Determinants of Fertility Distress.

Factor	Impact on Fertility	Clinical Implications
Age	Declining oocyte quality/quantity; Increased risk of endometriosis	Age-specific fertility preservation counseling; Early assessment of reproductive health
Tumor Type	Reproductive organ cancers: Direct structural damage Other cancers: Indirect gonadotoxic effects	Tumor-specific fertility preservation protocols; Fertility-sparing surgery when feasible
Treatment Modality	Surgery: Organ loss → infertility Chemotherapy: Gonadal suppression (e.g., cyclophosphamide) Radiotherapy: Ovarian dysfunction	Pre-treatment fertility preservation counseling; Oncofertility referrals prior to therapy initiation

**Table 2:** Psychosocial Drivers of Fertility Distress.

Domain	Key Findings	Intervention Opportunities
Psychological State	Higher anxiety/depression in fertility preservation patients; Resilience inversely correlates with fertility distress	Routine mental health screening; Cognitive-behavioral therapy in fertility preservation pathways
Social Support	Spousal/family support reduces fertility distress; Partner conflict increases anxiety	Couples counseling; Peer support networks
Socioeconomic Status	Higher SES → better care access; Financial barriers in LMICs → adverse outcomes	Financial navigation services; Policy advocacy for subsidized fertility preservation care
Medical Access	Telehealth enhances patient engagement; Resource gaps → inadequate counseling	Digital health platforms; Mobile fertility preservation clinics for underserved regions

SES, Socioeconomic Status; LMICs, low- and middle-income countries.

**Table 3:** Cultural Perspectives on Fertility.

Cultural Context	Core Beliefs	Impact on Fertility Preservation Decisions
Traditional Societies (Asian, African)	Fertility = “Divine duty”; Childlessness = social stigma	Guilt-driven fertility preservation pursuit; Delayed cancer treatment for fertility goals
Western Societies	Fertility = personal choice; Career prioritization	Greater openness to ART; Strategic childbearing delays
Globalized Settings	Hybrid values: Familial expectations + individual autonomy	Negotiated FP timelines; Increased ART utilization (e.g., IVF)

ART, Assisted Reproductive Technology; IVF, In Vitro Fertilization.

**Table 4:** Future Research Priorities.

Research Focus	Key Strategies	Expected Outcomes
Digital Interventions	AI-driven remote monitoring; VR counseling platforms	Improve patient adherence; Reduce Fertility distress severity
Long-term Survivorship	10-year QoL tracking post-fertility preservation; Reproductive autonomy metrics	Evidence-based fertility preservation guidelines; Policy reform catalysts
Integrative Frameworks	Combining biological/psychosocial/cultural assessments	Personalized fertility preservation pathways

AI, Artificial Intelligence; VR, Virtual reality; QoL, Quality of Life.

## Data Availability

Not applicable.

## Abbreviations

LMICs = low- and middle-income countries; PCOS = polycystic ovary syndrome; ART = Assisted Reproductive Technology; IVF = In Vitro Fertilization; AI = Artificial Intelligence; VR = Virtual reality; QoL = Quality of Life.
